# Fermented Vegetables and Legumes vs. Lifestyle Diseases: Microbiota and More

**DOI:** 10.3390/life13041044

**Published:** 2023-04-19

**Authors:** Eliza Knez, Kornelia Kadac-Czapska, Małgorzata Grembecka

**Affiliations:** Department of Bromatology, Medical University of Gdańsk, Gen. J. Hallera Aw. 107, 80-416 Gdansk, Poland; eliza.knez@gumed.edu.pl (E.K.); kornelia.kadac@gumed.edu.pl (K.K.-C.)

**Keywords:** fermented vegetables, lifestyle diseases, obesity, microbiome, probiotic food, fermentation, salt, antioxidants, short-chain fatty acids

## Abstract

Silages may be preventive against lifestyle diseases, including obesity, diabetes mellitus, or metabolic syndrome. Fermented vegetables and legumes are characterized by pleiotropic health effects, such as probiotic or antioxidant potential. That is mainly due to the fermentation process. Despite the low viability of microorganisms in the gastrointestinal tract, their probiotic potential was confirmed. The modification of microbiota diversity caused by these food products has numerous implications. Most of them are connected to changes in the production of metabolites by bacteria, such as butyrate. Moreover, intake of fermented vegetables and legumes influences epigenetic changes, which lead to inhibition of lipogenesis and decreased appetite. Lifestyle diseases’ feature is increased inflammation; thus, foods with high antioxidant potential are recommended. Silages are characterized by having a higher bioavailable antioxidants content than fresh samples. That is due to fermentative microorganisms that produce the enzyme β-glucosidase, which releases these compounds from conjugated bonds with antinutrients. However, fermented vegetables and legumes are rich in salt or salt substitutes, such as potassium chloride. However, until today, silages intake has not been connected to the prevalence of hypertension or kidney failure.

## 1. Introduction

In the XXI century, epidemics of lifestyle diseases can be observed. These are disorders mostly resulting from a sedentary lifestyle and an unhealthy diet, rich in simple sugars and saturated fats. Obesity is the most common noncommunicable disease, which can lead to many dangerous conditions such as hypertension, atherosclerosis, diabetes mellitus, and dyslipidemia. Those disorders may also occur as a result of an unhealthy lifestyle without the presence of obesity. Lifestyle diseases account for more than 70% of early deaths worldwide. The undisputed major cause of this phenomenon is obesity [1]. This condition is defined by the Food and Agriculture Organization (FAO) of the United Nations and the World Health Organization (WHO) as an excessive fat accumulation, and is diagnosed when Body Mass Index (BMI) is equal to or greater than 30 [1].

Not only obesity but also an unhealthy diet is one of the leading causes of the development of lifestyle diseases [2,3]. The Western Diet (WD) is rich in simple sugars and poor in dietary fiber, vegetables, and fruits. This type of nutrition is connected to elevated inflammatory markers, such as C-reactive protein (CRP), and is thought to initiate the activation of inflammation [4]. Changes in a daily menu that increase the consumption of fiber, fruits, and vegetables and decrease the intake of saturated fats and simple sugars are considered protective against lifestyle diseases. The most popular anti-inflammatory type of nutrition is the Mediterranean Diet (MD) [5,6]. However, there are specific food products that are highlighted by their pleiotropic health-promoting effect over the risk connected to excessive consumption of them. These are fermented products [7,8]. Silages show influence on gut microbiome diversity. These modifications exhibit an influence on the production of pro- or anti-inflammatory cytokines and affect gene level expression or enzyme activity [9].

The aim of this article was to summarize current knowledge concerning silages and find connections between fermented vegetables, their metabolism in the body, and related health complications. In the following sections, pickled vegetables and legumes and their influence on selected lifestyle diseases, including metabolic syndrome, obesity, non-alcoholic fatty liver disease (NAFLD), and diabetes mellitus, were described. Moreover, alternations in microbiota caused by these products and their impact on noncommunicable disorders were marked. Those products were chosen because there was a lack of information linking them to lifestyle diseases. Fermented dairy, fruits, cereals, and others were omitted. Other noncommunicable disorders, such as cancer, were omitted due to various changes in the human organism caused by a specific type of the disease (e.g., differences in gut microbiota). Moreover, the mechanisms by which these foods affect health were marked. The food products that were investigated are summarized in Table 1.

## 2. Methods

The analysis of the current state of knowledge about fermented vegetables and legumes was based on articles found in international databases: Scopus, ScienceDirect, and PubMed. The following expressions were used as search terms: “fermented vegetables”, “fermented legumes”, “microbiome”, “lifestyle diseases”, “obesity”, “metabolic syndrome”, “hypercholesterolemia”, “hypertension”, “NAFLD”, and “salt”. The most relevant literature sources were compiled in this review. Priority was given to articles published during the last five years.

## 3. Probiotic and Prebiotic Potential

### 3.1. Microbiome and Microbiome-Related Lifestyle Diseases

Gut microbiota are an integral part of homeostasis [28]. Microorganisms are able to produce and release many substances. Those can be short-chain fatty acids (SCFA), which exhibit an anti-inflammatory effect, immune cells (interleukins), and neurotransmitters (serotonin, dopamine). Noncommunicable diseases constitute a group characterized by microbiota alternations [29]. Changes in gut microorganisms’ diversity present in obesity compared to healthy control are the best known from lifestyle diseases. Most of them are repeatable. One of the characteristic features is a lower abundance of *Bacteroides* and *Prevotella* among obese people than in a healthy population [30]. Moreover, it is considered that targeted changes in the microbiota (e.g., through probiotic therapy) may be helpful in the treatment of obesity or diabetes mellitus [31].

Microbiota play an important role in energy expenditure. It was noted that *germ-free* mice have a lower body weight than conventional animals. It may be connected to the fermentation of nutrients in the intestines. Those compounds were called prebiotics, which are a source of energy for microorganisms, and which the human body does not digest. Therefore, those substances provide no energy value without microbiota. However, during the fermentation of prebiotics (e.g., fiber) by microorganisms, new compounds are formed. Most of them are SCFA, of which, 90% are absorbed by the colon and contribute up to 10% of daily energy value [29]. There is a link between obesity and increased SCFA and *Firmicutes* to *Bacteroidetes* ratio—the two general phyla capable of SCFA production [32]. Recent research confirmed that the concentration of SCFA is higher in obese patients than in people with BMIs below 30. Moreover, results showed that propionate was characterized by the greatest increase [33,34]. This is an important feature because this specific SCFA is a gluconeogenic substrate [35]. However, some research showed contradictory results and proved the positive influence of SCFA on energy expenditure [36,37]. Moreover, microbiota diversity is an important factor in lipid and glucose metabolism. It was noted that an increase in the Shannon index (diversity) and a greater number of *Firmicutes* and *Fusobacteria* are negatively correlated with LDL cholesterol [38]. Higher relative abundance of *Akkermansia muciniphila* results in improved glucose tolerance and reduced white adipose tissue (WAT) inflammation in obesity [39]. This condition is possibly connected to the higher gut permeability characteristic for gut dysbiosis present in obese people. This results in a higher concentration of lipopolysaccharides (LPS) in the blood–bacterial membrane molecules, which exhibit strong proinflammatory potential. These compounds were connected with increased weight gain, insulin resistance, and WAT inflammation [39]. These are features characteristic not only of obesity but also of diabetes, hypercholesterolemia, NAFLD, and cardiovascular disorders [40,41]. Moreover, obesity and the microbiota of obese patients may be the cause of the development of other metabolic disorders. That was proven in mice, where the microbiome of obese animals transplanted into *germ-free* mice showed NAFLD-like changes [42].

The human microbiome is subjected to increasingly advanced studies. There is a possibility that the shape of the gut microbiota is the most important factor forming the body, brain, and health. Therefore, maintaining a healthy microbiome may be a health determinant, including the prevention of lifestyle diseases.

### 3.2. Fermented Vegetables and Gut Microbiota

According to the International Scientific Association for Probiotics and Prebiotics (ISAPP) “fermented foods could benefit health through the nutritive alteration of the ingredients, modulation of the immune system, the presence of bioactive compounds that affect intestinal and systemic function, or by modulating gut microbiota composition and activity” [43]. According to the experts from FAO/WHO, the definition of probiotic is as follows: “Live microorganisms that, when administered in adequate amounts, confer a health benefit on the host” [44]. According to the European Food Safety Authority (EFSA), products claiming the presence of probiotic bacteria at the moment of consumption should contain a minimum of 10^6^ to 10^7^ cell forming units (CFU) per milliliter of viable bacteria [45,46]. The probiotic effect of fermented products was achieved despite the low viability of microorganisms in the gastrointestinal tract caused by environmental factors, such as low pH in the stomach. Moreover, the probiotic potential of pickled products depended on the microorganisms present in them that were able to influence microbiota diversity [47,48].

Lactic acid bacteria (LAB) are considered to be of key importance as probiotic microorganisms. That is due to their ability to produce lactic acid. Among LAB, *Lactobacillus, Pediococcus*, *Enterococcus*, *Leuconostoc*, *Streptococcus,* and *Lactococcus* can be distinguished [9]. Lactic acid bacteria were identified in most fermented vegetables, e.g., kimchi [49,50,51], sauerkraut [52,53], pickled cucumbers [54], and soy-based products [55]. The concentration of lactic acid is increasing along with the prolonged fermentation of vegetables [56]. As the best starter bacteria and lactic acid producers, *Lactiplantibacillus plantarum* and *Lactiplantibacillus fermentum* are considered [57]. That organic acid and all LAB exhibit a pleiotropic effect, including an increase in the bioavailability of macro- and micronutrients and an influence on appetite modulation and weight management [9]. It was confirmed that consumption of fermented vegetables increased LAB in feces and the concentration of lactic acid in the intestines [58].

*Bifdobacterium longum* is considered to be the bacterial strain that is known to exert antiobesity effects [59]. Moreover, it shows a lipid- and cholesterol-lowering effect in high-fat diet-induced obese rats [60]. An increase in intestinal *Bifidobacterium longum* improved metabolic disorders and reduced insulin resistance [61]. Moreover, supplementation of *Bifidobacterium longum* among obese women decreased the concentration of acetate and propionate in feces. Those are SCFAs positively correlated with obesity [62]. A fermented vegetable rich in *Bifidobacterium longum* is kimchi. Consumption of that Asian dish for eight weeks affected the diversity of gut microbiota in obese Korean women in the opposite direction from the microbiota characteristic of obesity and increased *Bifidobacterium longum* [63].

As it was said before, the predominant bacteria genera in fermented vegetables are LAB, and the type of fresh product determines their composition and quantity. However, there are several patterns that influence the microbial diversity of silages. Firstly, there is temperature and the pasteurization process. Silages heated over 72 °C were characterized by having zero living bacteria strains. Therefore, pasteurized fermented vegetables probably have no probiotic potential. However, there is a need for research in order to confirm or deny that hypothesis. Another factor affecting the microbial diversity of silages is salinity. The concentration of sodium chloride (NaCl) up to 3%, but not higher, resulted in the greatest microbial diversity, where the predominant genera were *Weisella* and *Lactobacillus* in pickled radish [64]. The concentration of salt 0.5%–10% provided LAB growth and microbiological safety [65]. However, fermented vegetables may be contaminated by pathogenic microorganisms such as *Listeria monocytogenes* or *Staphylococcus aureus*. The presence of these pathogenic organisms in silages was attributed mainly to poor handling and production practices and the native microflora of fresh foods [66].

There is still not enough research connected to fermented vegetables and the changes in gut microbiome caused by their consumption. It is important to gain knowledge of what modifications in microbiota diversity are due to silages intake. That information would help to understand the significant importance of fermented foods for intestinal microorganisms.

## 4. Epigenetic Changes

It was confirmed that alternations in microbiota diversity have an impact on gene levels of expression by up- or down-regulation [67,68,69]. That phenomenon may be important in the development and treatment of lifestyle diseases [70].

Higher concentrations of short-chain fatty acids (especially propionate) may induce obesity and disturb lipid metabolism due to G-protein coupled receptor (GPR) 41 and 43 upregulation. Greater adipogenesis is a result of it [71,72]. However, GPR 41 and GPR 43 induced peptide YY (PYY) and glucagon-like peptide 1 (GLP-1) release. Those are neurotransmitters that decreased appetite and fat accumulation and increased insulin sensitivity [73]. Moreover, a greater quantity of butyrate in the colon upregulated acyl-CoA synthetase long-chain family member 1 (ACSL1), thereby promoting catabolic processes such as β-oxidation and inhibiting lipogenesis [74]. Consumption of fermented kimchi had an impact on gene expression levels with a wide range of functionalities. ACSL1 was upregulated in the fermented kimchi group. ACSL1 is a trigger for activation of AMP-activated protein kinase (AMPK), thus inhibiting of biosynthesis of fatty acids and lowering blood pressure [63].

To conclude, type of nutrition should have an influence on greater production of butyrate, but probably not propionate, in order to decrease the risk of obesity and disturbed lipid and glucose metabolism. There is a need to perform numerous studies to unify this knowledge. Most research confirms the positive effects of SCFA, but due to contradictory results, further research is needed to confirm (or deny) this hypothesis. SCFA are probably the most important metabolites of the microbiota. There are numerous studies analyzing their influence on health. However, that knowledge is still disordered. There is a need to create a fine meta-analysis that concludes the risks and benefits associated with high SCFA concentration in the intestines.

## 5. Bioactive Peptides

Bioactive peptides (BPs) are specific protein fragments with various amino acid compositions and sequences. They exhibit beneficial effects on humans’ health, depending on their structural properties. Those compounds are naturally generated in foods through proteolysis carried out by endogenous or microbial enzymes during, e.g., the fermentation process [75]. Bioactive peptides demonstrate various biological functionalities, including antidiabetic, antihypertensive, antithrombotic, and antioxidant [76]. Most BPs are formed from milk protein [77]. However, soybeans are also a great source of these compounds [78]. Lactic acid bacteria, such as *Lactococcus lactis* or *Lactobacillus helveticus*, were characterized as the best starter cultures for BPs production in fermented foods. In the following sections, BPs formed during the fermentation of vegetables and legumes are described.

### 5.1. Inhibitors of Angiotensin I-Converting Enzyme

Inhibitors of angiotensin I-converting enzyme (ACE) are bioactive substances present in fermented foods [79]. Those compounds are peptides, such as isoleucine-proline-proline, valine-proline-proline, and leucine-proline-proline. These ingredients are used as nutraceuticals in hypertension treatment [80].

Bioactive peptides with ACE-inhibitory activity can be isolated from fermented vegetables. Due to the highest amount of protein in fresh samples, fermented legumes are characterized by a greater quantity of BPs than other vegetables [81]. Consumption of these products was associated with various health benefits. Fermented soybean condiments could attenuate diabetes-induced dyslipidemia that is considered to be associated with their ACE-inhibitory properties [82]. Fermentation of red beans by *Cardypes militaris* increased the number of essential amino acids and improved the in vitro protein digestibility of the legume. The pickled red bean showed ACE-inhibitory activity with an IC_50_ value of 0.63 mg protein/mL [83].

However, a link between fermented dairy products and inhibiting the renin-angiotensin-aldosterone system was not found [84]. More studies are required in order to unquestionably confirm or deny the activity of ACE inhibitors from food origins, including fermented vegetables.

### 5.2. Γ-Aminobutyric Acid

**Figure 1:**
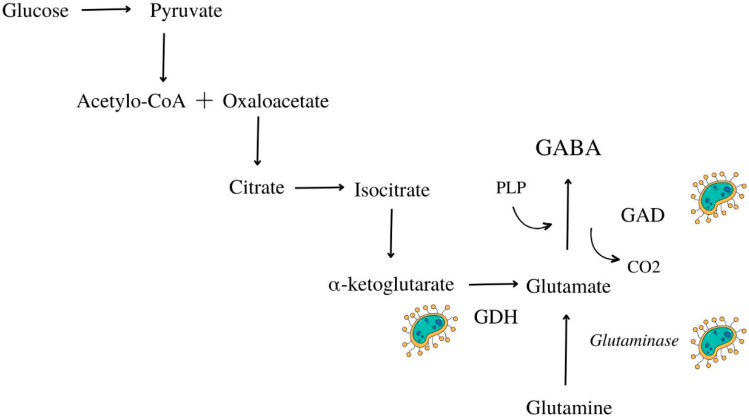
Metabolic pathway of GABA—production from glucose and glutamine by microbial enzymes. GABA—γ-aminobutyric acid, GAD—glutamate decarboxylase, GDH—glutamate dehydrogenase.

Γ-aminobutyric acid (GABA) is an important neurotransmitter with potential health benefits. In view of lifestyle diseases, it exhibits antihypertensive, antidepressant, and anticancer effects. Food studies showed that GABA could be supplied by the diet. It was found in fresh foods, but fermented products are distinguished by a higher amount of GABA. That is due to fermentative microorganisms capable of producing the glutamate decarboxylase (GAD) enzyme and proteolytic enzymes that decompose the amino acid glutamine (Figure 1) [85,86]. In several countries, γ-aminobutyric acid is classified as a dietary supplement (e.g., the United States), a medicinal ingredient (e.g., Canada), or an ingredient in food supplements (Europe). Some studies showed that GABA intake was associated with decreased blood pressure (approximately 10% of the initial value) [87]. The bioavailability of GABA from a food matrix (tomato puree) was investigated. In this study, alternations in the plasma concentration of GABA after oral ingestion were examined. Aqueous solution of GABA (888 mg GABA/L) and tomato puree (1044 mg GABA/L) constituted the study material. The average basic plasma concentration of GABA in men participating in the study amounted to 16 ng/mL. The plasma concentration of the analyzed compound after ingestion of tomato puree was higher than that of the aqueous solution (184 ng/mL vs. 74.7 ng/mL). These results suggest that food matrix may increase GABA absorption. The bioavailability was measured using the parameter area under the curve (AUC). The AUC of an aqueous solution was found to be 59.7, while tomato puree had an AUC of 115.7. An area under the curve higher than 100 may be connected to the presence of glutamate in food matrix. This compound is a precursor for GABA, which may have resulted in a higher plasma concentration of this compound. However, a maximum concentration (Cmax) of GABA was reached over a longer period of time in the case of food matrix than in aqueous solution [88]. This research showed that GABA could be supplied by foods rich in this compound.

Lactic acid bacteria are characterized by high efficiency in GABA synthesis [86,89]. This substance was found in pickled vegetables rich in LAB. Its presence was confirmed in cucumbers at a level of 1.32 mmol/L. The extracts from kidney beans fermented with *Lactiplantibacillus plantarum* exhibited potential antihypertensive activity due to their high γ-aminobutyric acid content (from 6.8 to 10.6 mg/g) [90]. Moreover, the concentration of GABA in fermented vegetables increases with prolonged fermentation. That was noted in kimchi. That is probably due to the greater number of LAB in silages. In addition, greater concentration of lactic acid provided adequate pH. Activity of GAD depends on acidity, and the pH for which this enzyme features the best productivity is between 4-5. However, accurate pH is conditioned by the predominant LAB produced GAD [85,91,92]. The final amount of GABA depends on particular fermentative strains. *Lactiplantibacillus plantarum* DSM19463 produces 4.83 mmol/L of GABA in 72 h but *Lacticaseibacillus paracasei* NFRI 7415–60 mmol/L in 144h [85]. There is still a lack of research on the bioavailability of GABA and glutamate from fermented vegetables and legumes. Due to their high content in these products, research concerning the absorption of GABA from silages should be performed.

Silages are considered unsuitable for hypertensive patients due to their high content of NaCl. However, studies did not find a link between increased consumption of pickled products and hypertension [93], which might result from high concentration of GABA in fermented vegetables.

### 5.3. Other Food-Derived Bioactive Peptides

**Figure 2:**
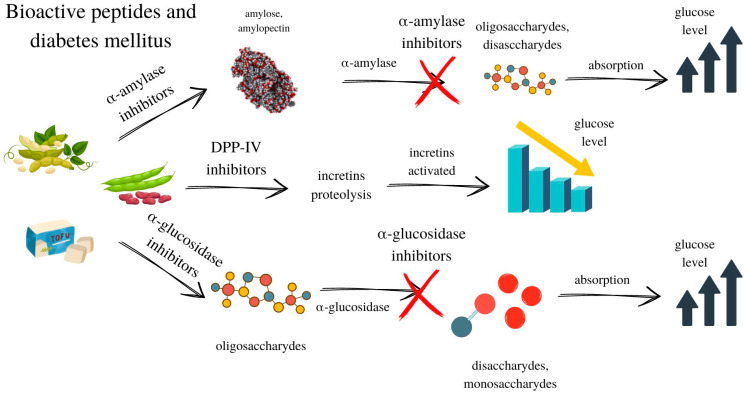
Mechanisms of bioactive peptides affecting glucose level based on [96]. Arrows indicate the direction of changes.

Bioactive peptides have been reported to show efficacy in modulating starch digestion and glucose absorption, such as the inhibition of α-glucosidase, α-amylase, and dipeptidyl peptidase IV (DPP-IV) (Figure 2). Moreover, peptides from plants can have a similar amino acid sequence to that of bovine insulin, as was proven for cowpea [94]. As with GABA and ACE inhibitors, legumes are characterized by the highest amount of other bioactive peptides of all vegetables due to the high concentration of proteins in these products [95]. Bioactive peptides were isolated from fresh legumes and leafy vegetables [95]. There is a lack of research concerning the influence of fermentation on the amount of these BPs. However, it can be assumed that their concentration in fermented vegetables and legumes would be higher than in fresh samples. That is due to enzymatic hydrolysis, conducted by microorganisms, which is the main process for obtaining peptides from their parent proteins and allowing them to have bioactivity. However, there should be conducted studies confirming this hypothesis.

## 6. Antioxidant Potential

Specific processing methods may increase antioxidants’ bioavailability in food products. Fermentation by microorganisms is characterized by such effects.

### Antioxidants Content in Fermented Vegetables

It was confirmed that fermented foods may decrease inflammation [97]. However, there is still limited research on silages. Most research was focused on fermented dairy products [97]. Due to the inflammation that occurs in obesity and other metabolic disorders, the antioxidant potential of fermented vegetables is one of their most important features. During the fermentation process, new compounds are formed, some of which have bioactivity. Moreover, silages are characterized by higher antioxidant potential than fresh samples [98]. This is due to the increased concentration of bioavailable phenols and other antioxidant compounds. In this section, components occurring in fermented vegetables that may be supportive factors in decreasing the risk of the development and treatment of lifestyle disorders are described.

The antioxidant potential of food products depends on the quantity and bioavailability of the compounds from the group of phenols, polyphenols, flavanols, and vitamin C. The influence of fermentation on the concentration of these substances in plants is mostly dependent on the fresh sample used, i.e., the type of vegetable (Table 2). However, several patterns can be recognized. A characteristic feature of pickled products is their greater bioavailability of phenols, polyphenols, and flavanols. This is due to LAB participating in the fermentation process and synthesizing the enzyme β-glucosidase, which is responsible for a decreased quantity of antinutrients located in complexes with antioxidant compounds [98]. Several vegetables are characterized by the presence of specific ingredients, such as glucosinolates (GLS) in *Cruciferous* plants [99]. These compounds are considered to be antinutrients. However, during fermentation, GLS are subjected to enzymatic degradation by mirosinase. Therefore, the breakdown products are formed. The main substances increased during fermentation from GLS breakdown are ascorbigen, indole-3-acetonitrile, indole-3-carbinol, and 3,3′-diindolylmethane. These components showed high antioxidant potential [23,100]. Nevertheless, there is research showing contradictory results and decreased levels of phenols and other antioxidant components (i.e., anthocyanins, carotenoids) [101,102]. Moreover, it was proven that the greater the number of phenolic compounds, the lower the concentration of LAB and lactic acid in fermented vegetables [103]. The divergent results are probably due to the various varieties and origins of specific vegetables. However, that is mostly because there is no standardized method to determine antioxidant potential [104]. It is impossible to come up with a solid statistic. There is an urgent need to create optimized and validated methods in order to obtain measurable results.

Fermentation of vegetables may have an adverse effect on the product’s composition. The most common unfavorable change after the fermentation of plant products is a decrease in vitamin C [105]. Salinity is an important factor affecting the concentration of that compound. It was shown that NaCl concentration may affect the level of vitamin C in the final product. A 2.5% NaCl concentration conditioned the lowest decrease in vitamin C (266.25 mg vitamin C/100 g vs. 156.27 mg vitamin C/100 g in 0.5% of NaCl). Therefore, that specific quantity of NaCl was responsible for the least loss of vitamin C in the final product [105]. Nevertheless, it was reported that a lower amount of vitamin C does not affect its general antioxidant potential [106].

There are many unanswered questions about vegetables’ antioxidant capacity. First of all, without a standardized method, results from different studies should not be compared. However, this is the only way to compile findings from various food products (Table 2). Antioxidant potential depends mainly on the freshness of the sample used (vegetable type). Most of the changes occurring during fermentation are beneficial, and in general, increased antioxidant potential can be observed. This is an important feature that makes these products valuable foods by helping decrease inflammation.

**Table 2:** Influence of fermentation on antioxidant compounds and antioxidant potential of vegetables and legumes.

Fermented Vegetable	Used Method	Identified Antioxidant Compound in Fermented Product	Influence of Fermentation on Antioxidant Compounds	Reference
Pigeon pea African yam bean Kidney bean	Spectrophotometric method	Polyphenols	Fermentation could increase the free soluble phenolic content and consequently enhance the antioxidant activities	[107]
Spinach	Spectrophotometric method	Polyphenols	Fermentation increases the phenolic content	[108]
Spinach Broccoli	Spectrophotometric method, LC-MS	Folic acid, 5-methyl tetrahydrofolate	Fermentation increases folic acid content	[109]
Chinese cabbage	Spectrophotometric method	Polyphenols, flavonoids	Fermentation increases phenolic content of the methanol extract and reduces flavonoid content in the water extract	[110]
Red cabbage	HPLC-DAD-MS/MS	Anthocyanins and derivatives of cyanidin (cyanidin-3-diglucoside-5-glucoside, cyanidin-3-(p-coumaroyl)-diglucoside-5-glucoside, cyanidin-3-(feruloyl)-diglucoside-5-glucoside, cyanidin-3-(sinapoyl)-diglucoside5-glucoside, cyanidin-3-(feruloyl)(feruloyl)-diglucoside-5-glucoside, cyanidin-3-(feruloyl)(sinapoyl)-diglucoside-5-glucoside, and cyanidin-3-(sinapoyl)(sinapoyl)-diglucoside-5-glucoside]	The diminution of anthocyanins content for the fermented product compared to the fresh product	[102]
Red cabbage	HPLC-MS/MS	Nonacylated and acylated anthocyanins with the main structure of cyanidin triglucoside	The diminution of anthocyanins content for the fermented product compared to the fresh product	[111]
Red beetroot	micro-HPLC-TOF-MS/MS	Betalains (betanin, isobetanin, betanidin and vulgaxanthin)	The fermentation of red beet reduced the content of betalains	[27]
Red beetroot	HPLC-MS/MS	Phenolic acids and flavonoids (isoferulic acid, protocatechuic acid, epicatechin, and apigenin	The fermentation process caused an increase in the content of free phenolic acids and reduced the content of conjugated phenolic acids The fermentation process caused a reduction in the content of free flavonoids and an increase in the content of conjugated flavonoids The fermentation process resulted in a reduction in the total content of phenolics (phenolic acids and flavonoids)	[112]

LC-MS—Liquid chromatography–mass spectrometry; HPLC—high-performance liquid chromatography; HPLC-TOF-MS/MS—high-performance liquid chromatography time-of-flight mass spectrometry; HPLC-MS/MS—high performance liquid chromatography and tandem mass spectrometry; HPLC-DAD-MS/MS—high-performance liquid chromatography with diode-array detection.

## 7. Salt

### 7.1. Salt and Salt Substitutes vs. Lifestyle Diseases

Salt, or sodium chloride (NaCl), is an inherent ingredient of life. It is mainly used to improve the taste of a meal and the microbiological safety of food products. Unfortunately, along with the development of the food industry, overconsumption of NaCl can be observed. The average person is exposed to great concentrations of NaCl in almost every food product on the market’s shelves. Daily intake of NaCl should not exceed 5 g (sodium itself should not be provided above 2 g) according to FAO/WHO recommendations, which can be difficult to do in the XXI century with ubiquitous processed foods [113]. Overconsumption of salt leads to many disorders, but hypertension is the most common result. In order to decrease NaCl intake, producers often use salt substitutes. These are mainly potassium chloride (KCl), calcium chloride (CaCl_2_), and monosodium glutamate. However, these substances may also have adverse effects on human health. In this section, the influence of salt and salt substitutes on selected lifestyle diseases is described.

Overconsumption of salt is considered an independent risk factor for obesity [114,115]. Increasing salt intake by 1 g per day was associated with a 28% increase in the risk of obesity [116]. Moreover, higher consumption of NaCl may also increase the risk of metabolic syndrome. This is due to the greater production of fructose. This substance, when administered to laboratory animals, can induce the metabolic syndrome [117]. Nevertheless, overconsumption of salt is mainly connected to hypertension. Reduced consumption of salt (less than 5 g per day) is recommended in the prevention and treatment of high blood pressure [118,119]. In addition, increased consumption of salt caused dysbiosis, a characteristic of hypertensive patients [120,121]. In recent years, KCl has become a popular NaCl substitute. It was proven that the replacement of 30% of NaCl by KCl in food products decreased the risk of hypertension development [122,123].

In XXI century, salt is ubiquitous. It is present in cereal products, dairy products, processed meat, and sweet and salty snacks, and it is used as an individual condiment by consumers. People should eat only unprocessed food in order to not exceed the recommended value. Otherwise, it is basically impossible. Fermented vegetables are another group of food products rich in salt. The influence of salty silages on human health was described in the following sections.

### 7.2. Salinity of Fermented Vegetables

Salt is one of the most important ingredients in fermented vegetables. Salt concentration has a significant effect on sensory, nutritional, and microbial quality and safety [124,125].

The sodium (Na) content of fermented food items in Korean products and its dietary intake were investigated. This macroelement was present in amounts ranging from 40 to 180 mg per serving size (100 to 450 mg NaCl). Specific amounts depended on the particular fermented product (various kimchi types). It was noted that the dietary intake of Na for an average Korean amounted to 698 mg (1745 mg of NaCl) only from fermented vegetables [124]. According to FAO/WHO, the daily intake of Na should not be greater than 2 g. That value was not exceeded in the cited study. However, that consumption was measured only for kimchi. Undoubtedly, this is not an exclusive source of food, even for Asian people. Salt is a ubiquitous food condiment, and its consumption may vary in different parts of the world. Still, fermented vegetables are not basic food products in most countries. They are used as additions to main dishes. Therefore, intake of salt from this source is probably a small part of the total NaCl consumption. However, due to the increasing popularity of silages all around the world, salt ingestion via fermented vegetables is also growing. There is an urgent need to perform research analyzing the daily intake of salt from other silages, not only kimchi, and report the results of the salt intake from other food products. Moreover, these studies should be conducted in various dietary cultures.

The composition of salty substances has an influence on the antioxidant potential of the final product. It was proven that the use of NaCl in Şalgam fermentation as the only flavor is characterized by an average amount of phenols (686 mg/L), while in the product where a mix of NaCl and CaCl_2_ was used, the concentration of these antioxidant compounds was the highest and amounted to 748.5 mg/L. For anthocyanins, the best composition of salty additive was NaCl with KCl (235.6 mg/L) or KCl with CaCl_2_ (238.4 mg/L). On the contrary, the lowest anthocyanins’ amount was when NaCl was used exclusively (205.3 mg/L) [126]. It can be assumed that the addition of other chloride salts, such as KCl or CaCl_2_ may improve the antioxidant potential of fermented vegetables. However, more research is needed in order to confirm this hypothesis.

0.5% of salt was a sufficient concentration to reduce sugar and accumulate more organic acid, and thus lowered pH and provided microbiological safety. That amount of NaCl allowed the best LAB growth and lactic acid production [65]. Moreover, a 0.5% salt concentration improved the sensory quality of sauerkraut [127]. The quantity of salt affects the diversity of microbiota in fermented vegetables. It was proven that most LAB (*Lactococcus* spp. and *Lactobacillus* spp.) had a relatively low abundance in the final product if the NaCl concentration was higher than 5%. On the contrary, *Pediococcus* spp. and *Weisella* spp. were characterized by their best growth when NaCl concentration was 10% [65,128]. It was found that the greater the NaCl concentration, the higher the pH and the lower the total acidity. However, that modification is considered to have no relevant effect [25]. Studies concerning the reduction of NaCl in fermented vegetables investigate only partial replacement by KCl or other salt substitutes. Adequate proportions in order to maintain microbiological safety, LAB growth, relevant acidity, and prevent spoilage of silages can reach 1:1 (NaCl:KCl). Moreover, such amounts allowed the studied food products to obtain the appropriate sensory qualities [129,130]. If NaCl constituted more than 50% of the salty additive, no adverse effect on nutritional or microbiological value was noted [126]. There is a need for studies analyzing the total replacement of NaCl by salt substitutes in fermented vegetables and their long-term durability.

Salt is an important ingredient in silages. However, there are still too many unknowns. The influence of salt concentration on silages’ quality is analyzed mainly with regard to soy products and kimchi. Other fermented vegetables, such as roots, are omitted. That is a mistake, because these products are gaining popularity among consumers all over the world. Depending on the culture, consumption of NaCl can be various; these should be included in research, especially as they concern hypertension prevalence and the consumption of silages. There is a need to conduct studies investigating the impact of salt and its substitutes on the nutritional, sensory, and microbial quality of all fermented vegetables. Moreover, the best recipe should be created using as little salt or its substitutes as possible, while maintaining adequate nutritional and sensory properties.

### 7.3. Salt, Fermented Vegetables, and Lifestyle-Diseases

In Section 7.1, the influence of salt on noncommunicable disorders is described. Most of the research on salt intake from fermented vegetables focuses on the hypertension risk due to the high salt concentration in them. Until today, the consumption of silages was not associated with hypertension prevalence. Moreover, consumption of silages may be connected to a decrease in blood pressure [131]. However, this research does not concern hypertensive patients. It was noted that kimchi intake for 8 weeks decreased systolic and diastolic blood pressure in overweight and obese patients [132]. There is a need for more research on the connection between silages intake and hypertension. It is necessary to gain knowledge about whether and in what amount patients with diagnosed hypertension may consume fermented vegetables.

Studies analyzing KCl intake from fermented vegetables also concern the prevalence of hypertension. It was noted that partial replacement of NaCl by KCl (30% KCl) is the best modification in order to decrease the risk of higher blood pressure [123]. However, there is a lack of research on hypertensive patients; therefore, medicine and doctors are not able to give fine recommendations to these people regarding silages consumption.

Beneficial influences on blood pressure and a decreased risk of hypertension related to the consumption of fermented vegetables may be connected to the bioactive peptides found in these products. Those are GABA and ACE inhibitors. However, no connection was found between peptides with ACE-inhibitory activity from fermented milk and changes in the renin-angiotensin-aldosterone system [84]. GABA, with regard to fermented foods, is considered to exhibit the desired effect [133]. There should be studies conducted concerning the intake of these substances and salt from the diet (e.g., fermented vegetables) and their impact on hypertension. The results should be compared with salt intake and the risk of hypertension. However, high blood pressure is not the only lifestyle disorder caused by the overconsumption of NaCl. Salt intake from silages needs to be contrasted with total diet and, other than hypertension, the prevalence of lifestyle diseases.

## 8. Fermented Vegetables and Legumes vs. Lifestyle Diseases

There are plenty of mechanisms by which fermented vegetables may affect health. Predominantly, it is a beneficial effect. However, silages may have an adverse impact on blood pressure and other lifestyle diseases due to the high concentration of salt in these products. Nevertheless, the consumption of fermented products was not connected to the prevalence of lifestyle disorders. Moreover, it may exhibit a positive influence on the prevalence of lifestyle diseases (Table 3). That is despite the high NaCl content or other salt substitutes. Research is still needed to confirm the beneficial effect of silages. Moreover, most studies focus on soy-based products and kimchi. This is a mistake, as those foods are the most popular in Asian countries but are less so in Europe or North America. In those regions, especially in Europe, sauerkraut and pickled cucumbers are characterized by the greatest popularity. Moreover, newer and newer silages are appearing on market shelves, such as fermented root vegetables. There is a need to investigate their influence on humans’ health.

## 9. Conclusions

Fermented vegetables are an important part of daily nutrition all over the world. After processing by microorganisms, they are characterized by various changes that differentiate them from fresh samples. These modifications mostly depend on the food product used for fermentation (e.g., type of vegetable). Still, several patterns can be highlighted. Fermented vegetables are characterized by a higher antioxidant potential due to the greater bioavailability of phenols. These compounds are released from conjugated bonds with antinutrients such as tannins and phytates, through the enzyme β-glucosidase produced by fermentative microorganisms. Moreover, during this kind of processing, proteins’ breakdown products are formed. These are short-chain amino acids that exhibit bioactivity (GABA, ACE-inhibitors).

One of the most popular and important features of fermented vegetables is their probiotic potential. It was repeatedly confirmed that consumption of silages affects the diversity of gut microbiota by increasing LAB in feces. There is a lack of research concerning how these changes affect patients with lifestyle disorders. However, knowing that microbiome changes occur in various lifestyle diseases, it can be assumed that modifications in microbiota diversity caused by consumption of fermented vegetables are beneficial in noncommunicable diseases. Nevertheless, research on lifestyle disorders and patients with these diseases should be conducted in order to confirm or deny these speculations.

Despite all the beneficial changes caused by silages, they may also have an adverse influence on humans’ health due to their high concentrations of salt and its substitutes. Nevertheless, until today, fermented vegetables intake was not related to hypertension or kidney damage prevalence. Unfortunately, there is a lack of studies investigating patients with diagnosed diseases. Moreover, previous research has analyzed mostly Asian people and the influence of kimchi. People who live in this region of the world are characterized by a relatively healthy diet based on plant products. There is a need to perform research in western countries, where WD and processed food dominate. Additionally, other fermented vegetables have to be investigated. These studies are very important for patients. Without them, doctors are unable to give these people fine recommendations concerning the permitted amount of silages to consume.

Fermented vegetables constitute a large open field of new research. There is also a need to determine the best fermentation conditions for obtaining the highest nutritional value of the final product. There are several patterns that can be recognized, such as the decrease in antinutrients during the process. However, considering the different effects of fermentation and its condition depending on the fresh sample used, more vegetables should be examined. Kimchi and fermented soy-based products are popular in Asian countries, and these foods are well known. There is an urgent need for deeper studies concerning vegetables consumed in other parts of the world.

## Figures and Tables

**Table 1:** Short characteristic of fermented food relevant for this review.

Food Product	Basic Material	Main Additives Different than Salt and Specific Fermentative Microorganisms	Source
Natto	Soybeans	*Bacillus subtilis natto*	[10]
Tempeh	Soybeans	*Rhizopus* spp.	[11]
Sufu (fermented tofu)	Soybeans	*Actinomucor elegans, Mucorracemosus, Mucor sufu, Mucor dispersus, Mucorracemosus, Mucor wutuongkiao,* and *Aspergillus* spp.	[12]
Soy sauce	Soybeans	Wheat flour/*Aspergillus oryaze, Tetragenococcus halophilus*, and *Zygosaccharomyces rouxii*	[13,14]
Doenjang	Soybeans	*Bacillus *subtilis**, *Bacillus. licheniformis*, *Bacillus megaterium, Aspergillus* spp., *Mucor* spp., and *Rhizopus* spp.	[15]
Chungkookjang	Soybeans	*Bacillus* spp.	[16]
Fermented legumes different than soya	Chickpeas, beans, peas, lentils, fava beans	LAB	[17,18]
Fermented cucumber	Cucumber	LAB	[19,20]
Kimchi	Chinese cabbage	Red pepper, garlic, ginger, leek, and glutinous rice flour/LAB	[21]
Sauerkraut	White cabbage	LAB	[22]
Fermented *Cruciferous* vegetables other than cabbage	Broccoli, cauliflower, brussels sprouts, kale	LAB	[23]
Fermented *solanaceous* vegetables	Tomatoes, eggplants, peppers (all kind)	LAB	[24,25]
Fermented roots	Carrots, radishes, beetroot, celery root, turnips	LAB	[26,27]

LAB—lactic acid bacteria.

**Table 3:** Influence of fermented vegetables on the prevalence of lifestyle diseases.

Fermented Product	Investigated Group	Effect	Source
Chungkookjang	Women and men with obesity and metabolic syndrome (n = 60), 12 weeks	Improvement in apolipoprotein B, which suggests potential antiatherosclerotic effect Decreased visceral fat (from 8.073 mm^2^ to 7.167 mm^2^) Decrease in LDL-C from 115.90 mg/100 mL to 111.21 mg/100 mL	[134]
Chungkookjang	Women and men with overweight/obesity (n = 166), 12 weeks	No significant difference in metabolic parameters between placebo and study group	[135]
Fermented soy product	Men with overweight and moderately increased cholesterol (n = 34), 12 weeks	Reduction of CVD risk markers in moderately hypercholesterolemic men by lipid profile improvement	[136]
Kimchi	Women and men with overweight (n = 21), 8 weeks	Decreased appetite, total energy intake, and body weight (2.1 kg) Decrease in systolic (from 126.5 mm Hg to 119.9 mm Hg) and diastolic (from 78.9 mm Hg to 74.9 mm Hg) blood pressure	[132]
Kimchi	Healthy volunteers (n = 100), 7 days	Decrease in systolic (from 120.3 mm Hg to 118.3 mm Hg) and diastolic (from 70.3 mm Hg to 69.3 mm Hg) blood pressure Decrease in TG (from 75.2 mg/dL to 67.7 mg/dL), TC (from 174.4 mg/dL to 165.5 mg/dL), and LDL-C (from 98.5 mg/dL to 91.7 mg/dL) Decrease in HDL-C (from 63.3 mg/dL to 60.7 mg/dL)	[137]
Doenjang	Overweight and obese patients (n = 83), 12 weeks	Decreased visceral fat Doenjang exhibited antiobesity and antioxidative effect due to improved PPAR-γ expression The catalase activity was increased	[138]

LDL-C—low density lipoprotein-cholesterol, CVD—cardiovascular diseases, TG—triglycerides, TC—total cholesterol, HDL-C—high density lipoprotein-cholesterol, PPAR-γ—peroxisome proliferator-activated receptors-γ.

## Data Availability

All data is contained within the article.
